# Feedback from Outcome Measures and Treatment Effectiveness, Treatment Efficiency, and Collaborative Practice: A Systematic Review

**DOI:** 10.1007/s10488-015-0710-5

**Published:** 2016-01-07

**Authors:** Dawid Gondek, Julian Edbrooke-Childs, Elian Fink, Jessica Deighton, Miranda Wolpert

**Affiliations:** Evidence Based Practice Unit, UCL and Anna Freud Centre, 21 Maresfield Gardens, London, NW3 5SU UK

**Keywords:** Feedback, Outcome measures, Outcome evaluation, Outcome management, Collaboratve practice

## Abstract

Due to recent increases in the use of feedback from outcome measures in mental health settings, we systematically reviewed evidence regarding the impact of feedback from outcome measures on treatment effectiveness, treatment efficiency, and collaborative practice. In over half of 32 studies reviewed, the feedback condition had significantly higher levels of treatment effectiveness on at least one treatment outcome variable. Feedback was particularly effective for not-on-track patients or when it was provided to both clinicians and patients. The findings for treatment efficiency and collaborative practice were less consistent. Given the heterogeneity of studies, more research is needed to determine when and for whom feedback is most effective.

**Electronic supplementary material:**

The online version of this article (doi:10.1007/s10488-015-0710-5) contains supplementary material, which is available to authorized users.

## Background

Feedback from outcome measures has become more widely used in mental health settings, as recent policy has placed increasing emphasis on the importance of using the views and preferences of patients to inform and guide practice (Carman et al. 2013). This has resulted in several recent studies being published (de Jong et al. 2012; Rise et al. 2012) after previous reviews examining the impact of feedback on treatment effectiveness (Carlier et al. 2012; Knaup et al. 2009). Thus, there is a strong need for an up-to-date systematic review synthesising and critically evaluating new studies. Providing a current account of evidence regarding the use of feedback may have benefits to clinical practice, as feedback has been found to enhance treatment effectiveness (Carlier et al. 2012; Knaup et al. 2009), particularly for not-on-track patients (Lambert et al. 2003). In addition, it may also contribute to treatment efficiency (Lambert et al. 2003) and collaborative practice (Jones and Delany 2014). Nonetheless, systematic evaluation of evidence regarding collaborative practice is lacking, meaning the present review is even timelier.

## Feedback Theories

Feedback from outcome measures provides clinicians and/or patients “with individual information on treatment outcome” based on outcome measures (e.g., mental health, symptom status, unmet needs) (Knaup et al. 2008, p. 15). The mechanisms by which feedback may benefit treatment outcomes are still largely unclear. The most commonly suggested mechanism of impact draws on Feedback Intervention Theory (Kluger and De Nisi 1996) and self-regulation theory (Scheier and Carver 2003). These theoretical frameworks suggest that if feedback is accepted by a clinician and/or patient as valid, a comparison is then made between actual and desired performance; for instance, between current progress and expected recovery. A discrepancy may motivate clinicians and/or patients to alter their behaviour, by for instance re-formulating therapeutic goals (Carlier et al. 2012; Greenhalgh et al. 2013) or increasing adherence to treatment (Riemer et al. 2005). Discrepancy between actual and desired performance should mainly occur for patients who are not responding to treatment as expected and, therefore, these patients are expected to particularly benefit from feedback (de Jong 2014).

## Treatment Effectiveness

The most recent reviews of feedback have shown that it is associated with higher levels of treatment effectiveness (Carlier et al. 2012; Knaup et al. 2009). A review from Carlier et al. (2012) included randomised control trials conducted within mental health and physical health settings without any restriction on population. In most included studies, health care professionals and patients received written feedback relevant to patients’ progress from routine outcome measures. In more than half of the studies (63 %), the experimental group receiving interventions supported by feedback from outcome measures showed more positive treatment outcomes than the control group not supported by feedback.

Within this review, the majority (70 %) of studies in mental health settings (primarily outpatient clinics) found that feedback was associated with higher levels of treatment effectiveness on at least one outcome (Carlier et al. 2012). Knaup et al. (2009) conducted a meta-analysis focussing on psychiatric or psychotherapeutic settings. Overall, feedback was associated with higher levels of treatment effectiveness; however, benefits tended to be short-term (Knaup et al. 2009).

In another meta-analysis, results from three studies conducted in the same college counselling centre were combined (Lambert et al. 2003) and it was found that feedback resulted in significantly fewer deteriorated patients compared to those not receiving feedbacks, that is, patients who worsened by at least 14 points on the Outcome Questionnaire-45 from pre-treatment to post-treatment (Lambert et al. 2003). Evidence suggests that feeding back results from outcome measures may be particularly beneficial for patients who are identified as not responding to treatment as expected or those not-on-track (Lambert et al. 2003; Sapyta et al. 2005).

## Treatment Efficiency

Feedback may also enhance treatment efficiency. On the one hand, feedback may indicate that the (off-track) patient needs treatment of higher frequency or intensity, which may result in more therapy sessions in order to achieve planned recovery (Lambert et al. 2003). On the other hand, feedback may indicate that the (on-track) patient needs treatment of lower frequency or intensity, which may result in fewer therapy sessions in order to achieve planned recovery (Lambert et al. 2003). Therefore, treatment efficiency may look quite different for different patients, with the combination potentially resulting in a null effect.

## Feedback Moderators

Feedback from outcome measures may not be associated with higher levels of treatment effectiveness in all instances, and it is unclear which characteristics of feedback moderate its effectiveness. It has been suggested that feedback from outcome measures is associated with higher levels of treatment effectiveness if feedback is given to both clinicians and patients compared to clinicians only, as it may facilitate the relationship between clinician and patient and in turn, enhance treatment outcomes (Garfield 1994). This hypothesis was supported by a recent meta-analysis of the effect of feedback on treatment effectiveness (Knaup et al. 2009).

## Collaborative Practice

Feedback, when openly shared, may also improve collaborative practice, as it facilitates discussion between patients and clinicians about current progress, treatment goals, and therapeutic approaches (Rothwell et al. 1997; Michie et al. 2008). Patients receiving feedback regarding their treatment may have a better understanding of their condition as well as the care they receive, which may trigger more active involvement in decision making (Michie et al. 2008). It opens space for a greater involvement of patients and their families in treatment (Jones and Delany 2014), improving patient-clinician communication both in mental health and physical health settings (Rothwell et al. 1997), patient satisfaction, and patient experience across a range of health settings (Elwyn et al. 2012). Feedback has also been found to increase patient autonomy, self-confidence (Joosten et al. 2011; Thomson et al. 2007), and adherence to treatment recommendations (Desroches et al. 2011; Wilson et al. 2010). Feedback also benefits clinicians, as it helps them engage in thinking about patients and it provides them with a greater sense of professional identity (Michie et al. 2008). Importantly, the impact of feedback on collaborative practice has not been examined in a systematic review.

## Aims of the Present Research

The aim of the present research was to systematically review the most up-to-date evidence about the associations of feedback with: (1) treatment effectiveness, with a particular focus on not-on-track patients in the feedback condition vs. not-on-track patients in the no-feedback condition, (2) treatment efficiency, with a particular focus on not-on-track patients in the feedback condition vs. not-on-track patients in the no-feedback condition, (3) treatment effectiveness according to feedback recipient, with a particular focus on whether providing feedback to both clinicians and patients vs. clinicians only moderates the effect of feedback, and (4) collaborative practice.

These research aims address gaps in the current literature, including the lack of up-to-date accounts of evidence regarding the association between feedback and treatment effectiveness, particularly for not-on-track patients who should especially benefit from feedback. In addition, there is no recent review evaluating the impact of feedback on treatment efficiency and potential moderators of feedback. Finally, to the best of our knowledge, there has been no attempt to systematically review evidence on the association between feedback and collaborative practice.

## Method

### Search Strategy

The search was developed according to best practice guidelines (Centre for Reviews and Dissemination 2009; Higgins and Green 2011; Moher et al. 2009). It was conducted in October 2013 in electronic databases meeting the criteria of best practice (Centre for Reviews and Dissemination 2009) and included PsycINFO (1806-October Week 4 2013), PsycEXTRA (1908-October 21 2013), Medline (1946-October Week 3 2013), Health Management Information Consortium (HMIC; 1979 to October 2013), Social Policy and Practice, and the Cochrane Library. Trials registers (European Union Clinical Trials Register, The International Standard Randomised Controlled Trial Number Registration, the Cochrane Central Register of Controlled Trials, and the United Kingdom Clinical Research Network) and grey literature (Opengray, Basesearch, Google Scholar) were reviewed for any unpublished studies. The search was updated in September 2014 using the same criteria. To help identify search terms, the research question was divided into two concepts: (a) mental health and (b) feedback.

Terms for mental health were identified using the Diagnostic Statistical Manual IV (American Psychiatric Association, 2000) and V (American Psychiatric Association 2015) and the International Classification of Disease 10 (World Health Organization 1992). A diverse array of terms for mental illness (e.g., depression, schizophrenia, and phobia) and symptomatology (e.g., delusions, anger) were included in searches of keywords in titles and abstracts, in addition to subject headings or Medical Subject Heading (MeSH) terms (with ‘exploding’ used to include narrower terms).

Terms for the feedback from outcome measures concept were identified through scanning keywords of relevant studies (Carlier et al. 2012; Chen et al. 2013; Duncan and Murray 2012; Knaup et al. 2009; Marshall et al. 2006). Due to the diversity of terms, a broad search strategy of keywords in titles and abstracts was used, including ‘feedback’, ‘outcome evaluation’, and ‘outcome management’. Synonyms, abbreviations, and spelling variations were identified for both concepts and combined in the search using the ‘OR’ Boolean operator, with concepts combined using ‘AND’. All references were imported to and managed with Excel and EndNote. Hand searches were carried out using the reference lists of relevant reviews (Carlier et al. 2012; Knaup et al. 2009) and included studies. Finally, authors and experts in the field were also consulted.

### Inclusion and Exclusion Criteria

All included studies were: (1) published in English, (2) used controlled designs such as randomised (cluster, block, open, parallel) or non-randomised trials, (3) conducted within specialist (i.e. developed specifically to address mental health problems) mental health settings (both inpatient and outpatient), (4) involved feedback based on standardised measures, (5) assessed the impact of feedback, using outcome measures, on at least one type of outcome related to treatment effectiveness (patient`s mental health), treatment efficiency (number of sessions), or collaborative practice (e.g., treatment satisfaction, treatment alliance).

Feedback was defined as providing clinicians and/or patients “with individual information on treatment outcome” based on outcome measures (e.g. mental health, unmet needs) (Knaup et al. 2008, p. 15). Outcome measures should be completed at least twice, at the outset of treatment and some period thereafter for change to be assessed; feedback should be provided at least once for it to inform treatment. There were no restrictions regarding demographic characteristics of the population or presenting mental health problems. Both published and unpublished studies (e.g., dissertations) were included.

Studies were excluded if measures were used for diagnosis or screening, feedback was not provided or the trial did not intend to evaluate the effects of feedback from outcome measures on at least one type of outcome related to treatment effectiveness, treatment efficiency, or collaborative practice.

### Search and Screening (see Fig. 1 for Details)

Initially, 5433 publications were identified. After excluding duplicates, 4075 publications remained for screening. After initial screening of titles and abstracts, 3759 were excluded. For the remaining 317 publications, full texts were retrieved. After eligibility assessment of the full texts, 291 publications were excluded; most publications were excluded as they described theoretical approaches to outcome feedback (*n* = 184; see supplementary Table 2 for details). Additional publications retrieved as a result of hand searching (*n* = 8) were included, resulting in a final sample that comprised 34 publications. However, two of the citations reported findings on the same study (Slade 2008; Slade et al. 2008) and one publication (Byrne et al. 2012) reported findings of a follow-up of another study (Newnham et al. 2010). Thus, 34 publications corresponding to 32 studies constituted the final sample.

**Fig. 1:**
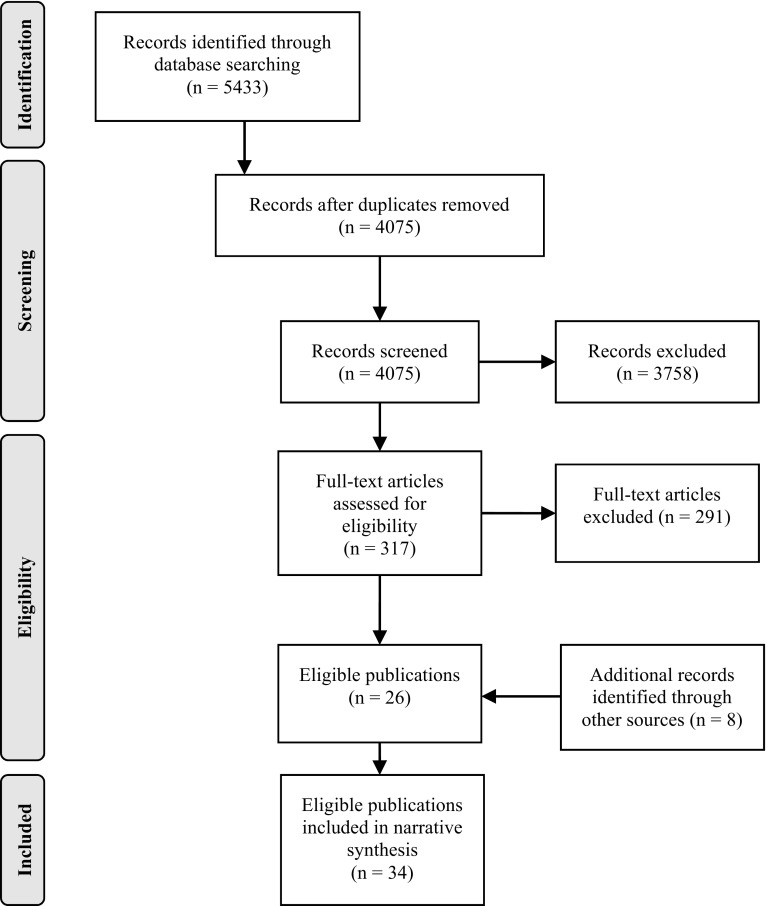
Flow diagram of study selection (adapted from Moher et al. 2009)

After removing duplicates, two authors (JEC and EF) screened titles and abstracts. If there was any possibility that a title and/or abstract could meet the inclusion criteria it was selected for further evaluation (‘low threshold’ strategy). The inter-rater reliability was high (Cohen’s kappa = .80). Full texts were retrieved for all citations, which were indicated as meeting the inclusion criteria by at least one of the authors. Unpublished or unavailable articles were retrieved with inter-library loans and by contacting the first two authors with two attempts per author. All full texts were then assessed by the first author (DG) with another author (JEC) assessing 20 %. Any disagreements were resolved by discussion.

### Data Extraction

Data were extracted from each included study using a data extraction form developed specifically for this review, drawing on best practice guidance (Centre for Reviews and Dissemination 2009; Higgins and Green 2011); the form was piloted on 20 % of included studies and refined. Extracted variables included authors, publication year, study design, type of publication, aim, location, illness, age of participants, gender, ethnicity, inclusion/exclusion criteria, recruitment process, unit of randomisation, method of randomisation, sample size, participants by condition, details of intervention (outcome measure administration and feedback), outcome measures, results, and analysis. Data were extracted from all full texts by the first author (DG) with another author (JEC) extracting data from 20 % of full texts to ensure consistency, and any discrepancies were resolved by discussion.

### Risk of Bias Assessment

The Cochrane Collaboration’s tool for assessing risk of bias was used for bias assessment (Higgins et al. 2011). All studies were assessed by the first author (DG) and the second author (JEC) evaluated 20 % of studies. In summary, the tool assesses the following types of biases as ‘high risk’, ‘low risk’, or ‘unclear risk’: (a) selection bias (random sequence generation and allocation concealment), (b) performance bias (blinding of participants and personnel), (c) detection bias (blinding of outcome assessment), (d) attrition bias (incomplete outcome data), (e) reporting bias (selective reporting), and (f) other biases.

### Synthesis of Results

Frequencies were used to summarise characteristics of individual studies, features of feedback interventions, and outcome measures used. Narrative synthesis (Popay et al. 2006) was used to compare the impact of feedback vs. no-feedback on: (1) treatment effectiveness, including measures of symptoms or general functioning (e.g., anxiety or depression), with an additional comparison of not-on-track patients in the feedback condition vs. not-on-track patients in the no-feedback condition; (2) treatment efficiency, including the number of sessions received, with an additional comparison of not-on-track patients in the feedback condition vs. not-on-track patients in the no-feedback condition; (3) treatment effectiveness depending on feedback recipient, including moderation effects of providing feedback to the clinician and patient vs. the clinician only; and (4) collaborative practice referring to the ‘process of care’, as conceptualised by Valderas and colleagues (2008), which includes patient-clinician communication, clinician behaviour (e.g., motivation, alliance with other professionals) and patient behaviour (e.g., motivation, treatment compliance). Satisfaction with treatment for both patient and clinician, and therapeutic alliance, were also included as collaborative practice outcomes.

## Results

### Characteristics of Included Studies (see Table 1 for Details)

More than half of included studies were published in North America (*n* = 19, 59 %). The majority of studies were published in peer-reviewed journals (*n* = 27, 79 %), with the remaining studies being doctoral theses (*n* = 7, 21 %). The sample size of included studies ranged from 24 to 3,919 participants. Most studies were conducted in outpatient settings (*n* = 26, 81 %), such as community-based mental health services (*n* = 22) and university counselling centres (*n* = 4); only six (19 %) studies were conducted in inpatient settings. A randomised control design was used in most of the studies (*n* = 29, 91 %). The diagnoses of patients varied greatly, including mood disorders, schizophrenia-like disorders, and eating disorders.

**Table 1:** Study Properties (adapted from Knaup et al. (2008))

Properties	*n*	*%*
Country		
North America	19	59
Germany	4	13
UK	3	9
Norway	2	6
Ireland	1	3
Australia	1	3
The Netherlands	1	3
Multicentre (Spain, the Netherlands, UK, Sweden, Switzerland)	1	3
Design		
Randomised control trial	29	91
Control trial	3	9
Publication type		
Published	27	79
Unpublished (dissertation)	7	21
Setting		
Out-patient	26	81
In-patient	6	19
Sample		
Adults	30	94
Adolescents	2	6
Unit of randomisation		
Patients	22	69
Professionals	3	9
Patients and professionals	3	9
Clinics	1	3
None	3	9

Most studies used no-feedback (*n* = 30, 94 %) as a comparison group; in seven studies (22 %) another experimental condition was also included. These other experimental conditions were: feedback vs. no-feedback, in which outcome measures were used but clinicians were instructed not to provide any feedback (Byrne et al. 2012; Newnham et al. 2010; Trudeau 2000); clinician feedback vs. clinician and patient feedback (Hawkins 2004; Priebe et al. 2007; Slade 2008; Slade et al. 2008); oral vs. written feedback (Galvinhill 2001); immediate vs. delayed feedback (Slade 2008; Slade et al. 2008); and feedback using different outcome measures (Copeland 2007). In two studies (6 %), alternative no-feedback condition were used, in which outcome measures were used but clinicians were instructed not to provide any feedback (Marshall et al. 2006) and clinician feedback vs. clinician and patient feedback (Cisneros 2010).

Treatment effectiveness was assessed most commonly with the Outcome Questionnaire-45 (OQ-45; *n* = 15, 47 %) and the Outcome Rating Scale (ORS; *n* = 6, 19 %), with the rest of the studies using a range of outcome measures assessing functioning (e.g., Symptoms and Functioning Severity Scale (SFSS); Bickman et al. 2011), psychosocial symptoms (e.g., Symptom Checklist-11; Brodey et al. 2005), quality of life (e.g., Subjective Quality of Life (SQOL); Priebe et al. 2007), wellbeing (e.g., World Health Organisation-Five Well-being Index (WHO-5); Byrne et al. 2012; Newnham et al. 2010), needs assessment (e.g., Cardinal Needs Schedule; Marshall et al. 2004), therapeutic processes (e.g., Helping Alliance Questionnaire; Cisneros 2010), and supervisees’ working alliance (e.g., Supervision Outcome Survey; Reese et al. 2009b).

### Feedback Characteristics (see Table 2 for Details)

Half of the studies used the OQ-45 (*n* = 16) to generate information provided in the feedback. In six (19 %) studies, researchers used the Session Rating Scale (SRS) or a combination of the SRS and the ORS. The other instruments used to generate information provided in the feedback included measures of psychosocial symptoms (e.g., Symptom Checklist-11; Brodey et al. 2005), functioning (e.g., SFSS; Bickman et al. 2011), wellbeing (e.g., WHO-5; Byrne et al. 2012; Newnham et al. 2010), quality of life (e.g., DIALOG; Priebe et al. 2007), needs assessment (e.g., Cardinal Needs Schedule; Marshall et al. 2004), and therapeutic processes (e.g., Empathy Scale; Copeland 2007).

**Table 2:** Study characteristics, feedback characteristics, and outcomes

Study characteristics				Feedback characteristics				Outcomes			
Reference	Sample (*N*)	Comparisons (*n*)	Outcome measures and follow-up period	Measures	Recipient	Frequency	Training provided	Treatment effectiveness: total sample	Treatment effectiveness: not on track patients	Treatment efficiency	Effect on collaborative practice
Anker et al. (2009)	Outpatient couple therapy (906)	Fb (446) NFb (460)	ORS, post-treatment, 6 months	SRS ORS	Clinician and patient	Session-by-session	Yes	Fb > NFb	N/A	N/A	N/A
Ashaye et al. (2003)	Outpatient (112)	Fb (44) NFb (58)	HoNOS 65 + , 3 months CAPE-BRS	CANE	Clinician	Once	Not reported	Fb = NFb	N/A	N/A	N/A
Bickman et al. (2011)	Outpatient community setting (340)	Fb (167) NFb (173)	SFSS, post-treatment	SFSS	Clinician	Session-by-session	Yes	N/A	N/A	Fb > NFb^a^	N/A
Brodey et al. (2005)	Outpatient (1374)	Fb (681) NFb (693)	SCL-11, 6 weeks	SCL-11	Clinician	Twice	Not reported	Fb > NFb	N/A	N/A	N/A
Cisneros (2010)	Outpatient (181)	Clinician-Fb (90) Patient-Clinician Fb (91)	OQ-45 ASC HAQ-II (all: post treatment)	OQ-45	Clinician/ Clinician and patient	Session-by-session	No	P/C-Fb > C-Fb	P/C-Fb > C-Fb	N/A	Therapeutic alliance P/C-Fb = C-Fb
Copeland (2007)	Outpatient (145)	NFb (39) LSQ Fb (37) ES Fb (33) Both Fb (35)	LSQ, sessions 1, 3, 5 ES-P and ES-N, sessions 2, 4	LSQ ES-P ES-N	Clinician	Session-by-session	Not reported	Fb = NFb	N/A	N/A	Therapeutic alliance Fb = NFb
De Jong et al. (2012)	Outpatient (413)	Fb (206) NFb (207)	OQ-45, after sessions 1, 3, 5, and then every fifth session	OQ-45	Clinician and patient	After sessions 1, 3, 5, and then every fifth session	Yes	Fb = NFb	Fb = NFb	N/A	N/A
Galvinhill (2001)	Outpatient Students (144)	NFb (48) Oral-Fb (48) Written-Fb (48)	OQ-45, post-treatment	OQ-45	Clinician	Session-by-session	Yes	O-Fb > NFb O-Fb > W-Fb W-Fb = NFb	N/A	N/A	N/A
Harmon et al. (2007)	Outpatient students (2819)	Clinician-Fb (687) Patient-Clinician Fb (687) NFb (1445)	OQ-45, post-treatment	OQ-45	Clinician/ Clinician and patient	Session-by-session	Not reported	Total Fb > NFb C-Fb = C/P-Fb	Total Fb > NFb C-Fb = P/C-Fb	N/A	N/A
Hawkins et al. (2004)	Outpatient (201)	Clinician- Fb (70) Patient-Clinician Fb (67) NFb (64)	OQ-45, post-treatment	OQ-45	Clinician/ Clinician and patient	Session-by-session	Not reported	C-Fb > NFb P/C-Fb > NFb P/C-Fb > C-Fb	C-Fb = NFb P/C-Fb = NFb C-Fb = C/P	Total Fb = NFb OT-Fb = OT-NFb NOT-Fb = NOT-NFb	N/A
Lambert et al. (2001)	Outpatient students (609)	Fb (307) NFb (302)	OQ-45, post-treatment	OQ-45	Clinician	Session-by-session	Not reported	Fb > NFb	Fb > NFb	Total Fb = NFb NOT-Fb < NOT-NFb OT-Fb > OT-NFb	N/A
Lambert et al. (2002)	Outpatient students (1020)	Fb (528) NFb (492)	OQ-45, post-treatment	OQ-45	Clinician	Session-by-session	Not reported	Fb > NFb	Fb > NFb	Total Fb < NFb NOT-Fb < NOT-NFb OT-Fb = OT-NFb	N/A
Lester (2012)	Inpatient (120)	Fb (60) NFb (60)	ORS, post-treatment Y-OQ, post-treatment	SRS ORS	Clinician and patient	Session-by-session	Yes	Fb = NFb	N/A	N/A	N/A
Marshall et al. (2004)	Outpatient (304)	Fb (158) NFb (146)	BPRS WHODAS CSI MANSA (all measures, 12 months)	CNS	Care coordinator	Once	Yes	Fb = NFb	N/A	N/A	Patient satisfaction Fb > NFb
Murphy et al. (2012)	Outpatient students (110)	Fb (59) NFb (51)	ORS, post- treatment	ORS	Clinician	Session-by-session	Yes	Fb = NFb	N/A	Fb = NFb	N/A
Newnham et al. (2010) (follow-up: Byrne et al. 2012)	Inpatient (1308)	Fb (408) NFb (439) Control (461)	WHO-5, 9 days SF-36, post-treatment DASS-21, post-treatment HoNOS, post-treatment	WHO-5	Clinician and patient	Twice	No	Fb = NFb	Fb > NFb	N/A	N/A
Priebe et al. (2007)	Outpatient (507)	Fb (256) NFb (235)	SQOL CSQ-8 PANSS (all: 12 months)	DIALOG	Clinician and patient	Every 2 months	Yes	Fb > NFb	N/A	N/A	Patient satisfaction Fb > NFb
Probst et al. (2013)	Inpatient (43)	Fb (23) NFb (20)	OQ-45, post-treatment	OQ-45	Clinician	Session-by-session	No	N/A	Fb > NFb	N/A	N/A
Probst et al. (2014)	Inpatient (209)	Fb (111) NFb (98)	OQ-45,1 week, 2 weeks, 3 weeks, post-treatment	OQ-45	Clinician	Session-by-session	No	Fb = NFb	N/A	N/A	N/A
Puschner et al. (2009)	Inpatient psychiatric (294)	Fb (148) NFb (146)	EB-45, T1.1, T1.2 (weeks after admission), T2 (discharge)	OQ-45	Clinician and patient	Session-by-session	Yes	Fb = NFb	N/A	N/A	N/A
Reese al. (2009a)	Outpatient (Study 1: 74) (Study 2: 74)	*Study 1* Fb (50) NFb (24) *Study 1* Fb (51) NFb (21)	ORS, post-treatment	SRS ORS	Clinician and patient	Session-by-session	Yes	Fb > NFb	N/A	Fb = NFb	N/A
Reese et al. (2009b)	Outpatient: community centre/students (110)	Fb (?) NFb (?)	ORS SOS SWAI–T COSE (all: T1- at the middle of the fall semester, T2 - end of the fall semester, T3 - at the end of the spring semester)	SRS ORS	Clinician and patient	Session-by-session	Yes	Fb > NFb	N/A	Fb = NFb	Supervisory alliance Fb = NFb Satisfaction with supervision Fb = NFb
Reese et al. (2010)	Outpatient couple therapy (92)	Fb (54) NFb (38)	ORS, post- treatment	OQ-45 SRS ORS	Clinician and patient	Session-by-session	Yes	Fb > NFb	N/A	Fb > NFb^a^	N/A
Reeves (2009)	Outpatient (220)	Fb (99) NFb (120)	OQ-45, post-treatment	OQ-45	Clinician and patient	Session-by-session	Yes	N/A	Fb = NFb	N/A	N/A
Rise et al. (2012)	Outpatient (75)	Fb (37) NFb (38)	TAS CSQ BASIS-32 PAM MCS (SF-12) Patient Motivation, Patient Activation, (all: 6 weeks)	SRS ORS	Clinician and patient	Session-by-session	Yes	Fb = NFb	N/A	N/A	Patient activation Fb = NFb Therapeutic alliance Fb = NFb Patient satisfaction Fb = NFb Patient motivation Fb > NFb
Simon et al. (2012)	Outpatient (370)	Fb (109) NFb (98)	OQ-45, post-treatment	OQ-45 ASC	Clinician and patient	Session-by-session	No	N/A	Fb > NFb	NOT-Fb = NOT-NFb	N/A
Simon et al. (2013)	Inpatient eating disorders (137)	Fb (69) NFb (64)	OQ-45, post-treatment	OQ-45	Clinician and patient	Session-by-session	No	Fb > NFb	N/A	N/A	N/A
Slade et al. (2008)/Slade (2008)	Outpatient (3919)	Clinician- Fb (687) Patient-Clinician Fb (687) NFb (1445)	OQ-45, post-treatment	OQ-45	Clinician/ Clinician and patient	Session-by-session	Not reported	Total Fb > NFb C = C/P	Total Fb = NFb C = C/P	NOT-Fb < NOT-NFb	N/A
Soeken et al. (1981)	Outpatient couple therapy (24)	Fb (?) NFb (?)	PSQ, T1, T2	PSQ	Clinician and patient	Session-by-session (sessions 6-10)	Not reported	Fb > NFb	N/A	N/A	Self-efficacy Fb > NFb Insight Fb > NFb Involvement Fb > NFb
Trudeau (2000)	Outpatient (127)	NFb (38) NFb (23) Fb (66)	RAND 36, 2 and 4 months OQ-45, 2 and 4 months	OQ-45	Clinician	Session-by-session	Not reported	Fb = NFb	N/A	N/A	N/A
Truitt (2011)	Outpatient eating disorders (51)	Fb (30) NFb (21)	OQ-45, post-treatment	OQ-45	Clinician and patient	Session-by-session	Not reported	Fb > NFb	N/A	Total Fb < NFb	N/A
Whipple et al. (2003)	Outpatient students (981)	Fb (499) NFb (482)	OQ-45, post-treatment	OQ-45	Clinician	Session-by-session	No	Fb = NFb	Fb > NFb	Total-Fb = NFb NOT-Fb < NOT-NFb OT-Fb > NOT-NFb	N/A

*Fb* feedback, *NFb* no-feedback, *ORS* Outcome Rating Scale, *SRS* Session Rating Scale, > significantly higher level, < significantly lower level, = no difference, *N/A* not applicable, *HoNOS 65*+ Health of the Nation Outcome Scales 65+, *CAPE-BRS* Clifton Assessment Procedures for the Elderly-Behaviour Rating Scales, *CANE* Camberwell Assessment of Need for the Elderly, *NS* not significant, *SFSS* Symptoms and Functioning Severity Scale, *CFS* Contextualized Feedback System, *SCL-11* Symptom Checklist-11, *LSQ* Life Status Questionnaire, *ES* Empathy Scale, *ES-P* Empathy Scale—Positive, *ES-N* Empathy Scale—Negative, *OQ-45* Outcome Questionnaire—45, *O-Fb* Oral Feedback, *W-Fb* Written Feedback, *C-Fb* Clinician Feedback, *C/P-Fb* Clinician/Patient Feedback, *OT* on-track, *NOT* not-on-track, *Y*-*OQ* Youth Outcome Questionnaire, *BPRS* Brief Psychiatric Rating Scale, *WHODAS* World Health Organization Psychiatric Disability Assessment Schedule, *CSI* Client Satisfaction Index, *MANSA* Manchester Short Assessment of Quality of Life, *CNS* Cardinal Needs Schedule, *WHO-5* World Health Organisation, *SF-36* Short Form 36 Health Survey, *DASS-21* Depression Anxiety Stress Scales, *ASC* Assessment for Signal Clients, *HAQ-II* Helping Alliance Questionnaire, *SQOL* Subjective Quality of Life, *CSQ-8* Client Satisfaction Assessment, *PANSS* Positive and Negative Syndrome Scale, *EB-45* Ergebnisfragebogen, *SOS* Supervision Outcome Survey, *SWAI–T* Supervisory Working Alliance Inventory, *COSE* Counselling Self-Estimate Inventory, *TAS* Treatment Alliance Scale, *CSQ* Client Satisfaction Questionnaire, *BASIS-32* Behaviour and Symptom Identification Scale, *PAM* Patient Activation Measure, *MCS* (*SF-12*) Mental Component Score (Short Form-12), *PSQ* Post Session Questionnaire, *RAND* RAND 36-item Health Survey 1.0

^a^Patients in the feedback condition improved significantly faster

Feedback was provided either to clinicians and patients (*n* = 15, 47 %) or clinicians only (*n* = 12, 38 %). In four (13 %) studies, conditions with both feedback recipients were used; in one case, the feedback recipient was the care coordinator (Marshall et al. 2004). In most cases, feedback was administered session-by-session (*n* = 26, 81 %), and in other studies it was provided twice (*n* = 2, 6 %) (Brodey et al. 2005; Byrne et al. 2012; Newnham et al. 2010), once (*n* = 2, 6 %) (Ashaye et al. 2003; Marshall et al. 2004), every 2 months (*n* = 1, 3 %), or in one study (3 %), at session 1, 3, 5, and then every 5th session (de Jong et al. 2012). Importantly, in studies where feedback was provided only once (Ashaye et al. 2003; Marshall et al. 2004), it included a detailed needs assessment and a list of suitable interventions to address these needs. Thus, the content of feedback in these two studies differed from interventions in which feedback was administered on a more frequent basis, where it was mostly limited to progress monitoring. Clinicians who provided feedback were not always given training in the use of feedback (*n* = 6, 19 %), and in twelve (38 %) studies this information was not reported. Feedback in most studies (*n* = 23, 72 %) included information about treatment progress; in seven (22 %) studies it was supported by additional feedback components such as a decision tree, a list of suitable interventions, or treatment recommendations.

### Risk of Bias Assessment (see Supplementary Table 1 for Details)

Most studies did not report enough detail to make a valid judgement regarding the presence of risk according to the Cochrane tool (Higgins et al. 2011).

#### Sequence Generation

In terms of sequence generation, twelve (38 %) studies provided sufficient information, three of which had a high risk of bias due to a lack of randomisation (Byrne et al. 2012; Lambert et al. 2002; Newnham et al. 2010; Reeves 2009). In addition, two (6 %) studies reported problems with randomisation, resulting in some clinicians being non-randomly assigned to conditions (Reese et al. 2009b) and a highly unequal distribution of patients across clinicians (Copeland 2007).

#### Allocation Concealment

A lack of randomisation also introduced a high risk of bias related to allocation concealment in three (9 %) studies (Byrne et al. 2012; Lambert et al. 2002; Newnham et al. 2010; Reeves 2009). Allocation concealment could have been a source of bias in two (6 %) other studies as patients were assigned to clinicians by the unit manager (Rise et al. 2012) and allocation was inconsistent across conditions (Reese et al. 2009b).

#### Blinding of Patients

Six (19 %) studies reported on the process of blinding patients, two of which were judged as having a high risk of bias as no attempt was made to blind patients (Puschner et al. 2009; Rise et al. 2012). None of the studies successfully blinded clinicians to feedback conditions.

#### Incomplete Outcome Data

One of the most prevalent risks of bias related to incomplete outcome data, and 11 (34 %) studies were judged as having a high risk, mostly due to high attrition (Brodey et al. 2005; Copeland 2007; de Jong et al. 2012; Galvinhill 2001; Lambert et al. 2001, 2002; Lester 2012; Probst et al. 2014; Probst et al. 2013; Reese et al. 2009a; Trudeau 2000). Only five (16 %) studies reported using intention-to-treat analysis to account for attrition (Bickman et al. 2011; Priebe et al. 2007; Puschner et al. 2009; Rise et al. 2012; Schmidt et al. 2006).

#### Selective Outcome Reporting

Selective reporting of outcomes was not a source of bias in any of the studies.

#### Other Sources of Bias

Finally, 18 (56 %) studies reported other potential sources of bias, where the most common reason (*n* = 14, 44 %) was a small sample size (Ashaye et al. 2003; Copeland 2007; Galvinhill 2001; Lester, 2012; Murphy et al. 2012; Probst et al. 2013; Reese et al. 2009a, b; Rise et al. 2012; Schmidt et al. 2006; Simon et al. 2013; Soeken et al. 1981; Trudeau 2000; Truitt 2011).

### Treatment Effectiveness (see Table 2 for Details)

Overall, 27 studies compared treatment effectiveness between feedback vs. no-feedback conditions. In more than half of these studies (*n* = 15, 56 %) patients in the feedback condition had significantly higher levels of treatment effectiveness than patients in the no-feedback condition on at least one treatment outcome variable (Anker et al. 2009; Brodey et al. 2005; Galvinhill 2001; Harmon et al. 2007; Hawkins 2004; Lambert et al. 2001, 2002; Priebe et al. 2007; Reese et al. 2009a; Reese et al. 2010; Reese et al. 2009b; Simon et al. 2013; Slade 2008; Slade et al. 2008; Soeken et al. 1981; Truitt 2011).

In 12 (44 %) studies there were no significant differences in treatment effectiveness between patients in the feedback vs. no-feedback conditions (Ashaye et al. 2003; Byrne et al. 2012; Copeland, 2007; de Jong et al. 2012; Lester, 2012; Marshall et al. 2004; Murphy et al. 2012; Newnham et al. 2010; Probst et al. 2014; Puschner et al. 2009; Rise et al. 2012; Trudeau 2000; Whipple et al. 2003). This included three studies with more than one feedback condition (Byrne et al. 2012; Copeland 2007; Newnham et al. 2010; Trudeau 2000).

#### Not-on-Track Patients

In 11 studies, additional comparisons were made regarding treatment effectiveness between not-on-track patients in the feedback vs. no-feedback conditions. In the majority of these studies (*n* = 8, 73 %) not-on-track patients in the feedback condition had higher levels of treatment effectiveness for at least one outcome variable than not-on-track patients in the no-feedback condition (Byrne et al. 2012; Harmon et al. 2007; Lambert et al. 2001, 2002; Newnham et al. 2010; Probst et al. 2013; Simon et al. 2012; Slade 2008; Slade et al. 2008; Whipple et al. 2003). In three studies there were no differences in treatment effectiveness between not-on-track patients in the feedback vs. no-feedback conditions (de Jong et al. 2012; Hawkins 2004; Reeves 2009).

### Treatment Efficiency (see Table 2 for Details)

Overall, 10 studies compared treatment efficiency between patients in the feedback vs. no-feedback conditions. In two (20 %) studies patients in the feedback condition had higher levels of treatment efficiency than patients in the no-feedback condition (Bickman et al. 2011; Reese et al. 2010). In six (60 %) studies there was no difference in treatment efficiency between patients in the feedback vs. no-feedback conditions (Hawkins, 2004; Lambert et al. 2001; Murphy et al. 2012; Reese et al. 2009a; Reese et al. 2009b; Whipple et al. 2003). In contrast, in two (20 %) studies patients in the feedback condition had lower levels of treatment efficiency than patients in the no-feedback condition (Lambert et al. 2002; Truitt, 2011).

#### On-Track vs. Not-on-Track Patients

In addition, six studies compared treatment efficiency between not-on-track patients in the feedback condition vs. not-on-track patients in the no-feedback condition. In four (67 %) studies not-on-track patients in the feedback condition had lower levels of treatment efficiency than not-on-track patients in the no-feedback condition (Lambert et al. 2001, 2002; Slade 2008; Slade et al. 2008; Whipple et al. 2003). In two (33 %) studies there was no difference in treatment efficiency between not-on-track patients in the feedback vs. no-feedback conditions (Hawkins 2004; Simon et al. 2012).

Four studies also included a comparison of treatment efficiency between on-track patients in the feedback condition vs. on-track patients in the no-feedback condition. In two (50 %) studies on-track patients in the feedback condition showed higher levels of treatment efficiency than on-track patients in the no-feedback condition (Lambert et al. 2001; Whipple et al. 2003), whereas in two studies there was no difference between conditions (Hawkins 2004; Lambert et al. 2002).

## Feedback Recipient

Feedback was provided only to clinicians in 12 studies (Ashaye et al. 2003; Brodey et al. 2005; Copeland 2007; Galvinhill 2001; Hawkins 2004; Lambert et al. 2001; 2002; Marshall et al. 2006; Murphy et al. 2012; Probst et al. 2014; Trudeau 2000; Whipple et al. 2003). In five (42 %) of these studies patients in the feedback condition had higher levels of treatment effectiveness than patients in the no-feedback condition for at least one outcome variable (Brodey et al. 2005; Galvinhill 2001; Hawkins 2004; Lambert et al. 2001, 2002).

In 14 studies feedback was given to patients and clinicians (Anker et al. 2009; Byrne et al. 2012; de Jong et al. 2012; Hawkins 2004; Lester 2012; Newnham et al. 2010; Priebe et al. 2007; Puschner et al. 2009; Reese et al. 2009a; Reese et al. 2010; Reese et al. 2009b; Rise et al. 2012; Simon et al. 2013; Soeken et al. 1981; Truitt, 2011). In nine (64 %) of these studies patients in the feedback condition had higher levels of treatment effectiveness than patients in the no-feedback condition for at least one outcome variable (Anker et al. 2009; Hawkins, 2004; Priebe et al. 2007; Reese et al. 2009a; Reese et al. 2010; Reese et al. 2009; Simon et al. 2013; Soeken et al. 1981; Truitt 2011). In addition, four studies directly compared clinician vs. clinician-patient recipient conditions. In two (50 %) studies patients in the clinician-patient condition had higher levels of treatment effectiveness compared to patients in the clinician-only condition (Cisneros 2010; Hawkins 2004), whereas in the two remaining studies a significant difference was not found (Harmon et al. 2007; Slade 2008; Slade et al. 2008).

### Collaborative Practice (see Table 2 for Details)

There were seven studies that compared the effect of outcome feedback on collaborative practice between patients in the feedback vs. no-feedback conditions. Patients in the feedback condition showed higher levels of satisfaction with treatment (*n* = 2; Marshall et al. 2004; Priebe et al. 2007), patient motivation referring to knowledge, skill, and confidence for self-management of their condition (Rise et al. 2012), self-efficacy (*n* = 1; Soeken et al. 1981), insight (*n* = 1; Soeken et al. 1981), and involvement in care (*n* = 1; Soeken et al. 1981) compared to patients in the no-feedback condition.

There was no difference between patients in the feedback vs. no-feedback conditions, in satisfaction with supervision and the supervisory relationship (*n* = 1; Reese et al. 2010), therapeutic alliance (*n* = 2; Copeland, 2007; Rise et al. 2012), level of patients’ activation (*n* = 1; Rise et al. 2012), and patients’ satisfaction (*n* = 1; Rise et al. 2012).

## Discussion

The aim of the present research was to systematically review evidence of the impact of feedback from outcome measures on treatment effectiveness, treatment efficiency, and collaborative practice. We examined whether there were differences due to feedback provision in terms of: (1) treatment effectiveness, with a particular focus on not-on-track patients in the feedback condition vs. not-on-track patients in the no-feedback condition patients; (2) treatment efficiency, with a particular focus on not-on-track patients in the feedback condition vs. not-on-track patients in the no-feedback condition; (3) treatment effectiveness depending on feedback recipient, with a particular focus on whether providing feedback to clinicians and patients vs. clinicians only moderates the effect of feedback; and (4) collaborative practice.

In more than half of these studies, patients in the feedback condition had significantly higher levels of treatment effectiveness than patients in the no-feedback condition on at least one treatment outcome variable, which was in line with findings from previous reviews (Carlier et al. 2012; Knaup et al. 2009). Feedback was found to be particularly beneficial for not-on-track patients, who had higher levels of treatment effectiveness than not-on-track patients in the no-feedback condition for at least one outcome variable in 73 % of the studies examining these groups. This finding dovetails with previous studies (Lambert et al. 2003) and theories explaining feedback mechanisms, such as the Feedback Intervention Theory (Kluger and De Nisi 1996) and self-regulation theory (Scheier and Carver 2003), which highlight the role of discrepancy between treatment goals and actual progress as the main drive for behaviour change. Feedback is theorised to not only trigger dissonance due to discrepancy between experienced and expected treatment progress—and consequently, corrective behaviour change—but it also improves patients’ insight into difficulties, whilst providing reassurance that treatment goals are achievable (Michie et al. 2008).

The findings regarding differences in treatment efficiency due to feedback provision were highly varied. Considering main effects, six out of ten studies showed no difference between the feedback and no-feedback conditions; two studies showed higher levels of efficiency in the feedback condition; and another two studies showed higher levels of efficiency in the no-feedback condition. Overall, feedback did not reduce the number of sessions received by patients, which was inconsistent with findings from the meta-analysis conducted by Lambert et al. (2003).

Additional comparisons showed that not-on-track patients in the feedback condition received more sessions than not-on-track patients in the no-feedback, as also found in the previous meta-analysis (Lambert et al. 2003). The results for on-track patients were mixed, with two studies showing higher levels of treatment efficiency in the feedback condition and two studies showing no difference between conditions, which was not in full agreement with the previous review showing that on-track patients received fewer sessions as a result of feedback (Lambert et al. 2003). Nonetheless, it is necessary to highlight that Lambert and colleagues (2003) included only three studies in their analysis, which were conducted by the same research team. Thus, the characteristics of feedback and studies were more homogeneous than in the present review, which may partially explain the discrepancy in findings.

Nonetheless, in nearly all studies in which patients in the feedback condition received a greater or equal number of sessions than patients in the no-feedback condition, patients also had higher levels of treatment effectiveness. This seems to be the case particularly for the not-on-track patients who were most consistently reported to stay in treatment longer but also experience higher levels of treatment effectiveness.

These findings are in line with theories of feedback, such as the Feedback Intervention Theory (Kluger and De Nisi 1996) and self-regulation theory (Scheier and Carver 2003). Regular use of outcome measures may indicate early in treatment that patients are not progressing as expected and feedback may facilitate any necessary adjustments to the treatment, for instance in terms of its intensity or duration. Patients in the feedback condition may have felt more informed about and involved in their treatment, resulting in a greater motivation to remain in therapy longer in order to reduce the discrepancy between treatment goals and actual improvement (Michie et al. 2008). Clinicians, in turn, may be more committed as a result of feedback to provide effective treatment even if requires a greater number of sessions.

In line with previous research, we found a high percentage of studies showing higher levels of treatment effectiveness when feedback was given to both clinicians and patients (64 %) than clinicians exclusively (42 %; Knaup et al. 2009). Such an effect may be explained by an improved relationship between patients and clinicians moderating treatment effectiveness (Garfield 1994). Nonetheless, studies directly comparing these conditions showed mixed results, where half of studies indicated higher levels of treatment effectiveness when feedback was given to both patients and clinicians. The remaining studies did not show a significant difference between conditions.

Finally, based on previous research (Valderas et al. 2008), we expected that outcome feedback would improve collaborative practice between patients and clinicians; however, the findings of the present review did not fully support this. It was particularly striking that feedback was not shown to improve therapeutic alliance (Cisneros 2010; Copeland 2007; Rise et al. 2012). These findings are contrary to the assumptions based on the literature that feedback contributes to building a collaborative therapeutic environment with the involvement of both patients and clinicians (Ackerman and Hilsenroth 2003). However, it is noteworthy that in Copeland`s (2007) study therapeutic alliance increased across all conditions causing a ceiling effect and, as explained by the authors, there may have been insufficient statistical power to detect significant differences between conditions. Similarly, Cisneros (2010) found therapeutic alliance to be universally high across conditions.

Still, it is important to note that feedback was found to have a positive effect on patients’ motivation by one study (Rise et al. 2012). In addition, patients’ satisfaction with the treatment was found to be greater in the feedback condition in two studies (Marshall et al. 2004; Priebe et al. 2007), whereas one study did not show a significant difference between the conditions; however as pointed out by the authors, the study may have been underpowered (Rise et al. 2012). Finally, one study that included multiple collaborative practice outcomes showed a positive effect of feedback on patients’ self-efficacy, insight, and involvement (Soeken et al. 1981). Thus, despite a possible lack of effect of feedback on therapeutic alliance, feedback appears to facilitate the relationship between clinicians and patients in other ways.

Overall, as stated by Valderas et al. (2008), the highly heterogeneous results in the extant literature do not allow for any robust inferences regarding the impact of outcome feedback on collaborative practice. Clearly, further empirical research is needed to examine the association between feedback and collaborative practice.

The growing emphasis on treatment that is characterised by greater patient involvement (Carman et al. 2013) has resulted in an increase in studies investigating the effect of outcome feedback in mental health practice. This current review provides a much-needed up-to-date account of evidence in the area, including recent studies that were not captured in previous reviews. In particular, there has been an increase in studies conducted outside of North America and the UK, which entirely dominated previous reviews (Carlier et al. 2012; Gilbody et al. 2001; Knaup et al. 2009; Valderas et al. 2008). Moreover, due to promising findings from studies conducted mainly in outpatient settings and with adult populations (Knaup et al. 2009), feedback interventions have been gradually spread across various mental health settings (e.g., psychiatric inpatient) and populations (e.g., couples, children, adolescents), which have not been featured in previous reviews (Carlier et al. 2012; Gilbody et al. 2001; Knaup et al. 2009; Valderas et al. 2008). In addition, the current study was informed by previous reviews, attempting to address their limitations and provide cumulative evidence on issues reported by the researchers as of high importance for clinical practice (Carlier et al. 2012; Gilbody et al. 2001; Knaup et al. 2009; Valderas et al. 2008). As a result, the current study focused on not-on-track patients, providing an insight into the effective use of feedback in terms of its recipient and presented an account of evidence regarding the impact of feedback on collaborative practice.

Nevertheless, limitations should be considered when interpreting the findings of the present review. As in previous reviews (Carlier et al. 2012; Valderas et al. 2008), the heterogeneity of studies with respect to sample, measures, and methodology made it challenging to synthesise findings and prohibited meta-analysis. Moreover, the comparison between feedback systems was problematic as they significantly differed across studies in terms of their characteristics (e.g., frequency, intensity, level of training provision). Findings from studies such as the in-progress trial examining the effectiveness of components of feedback (van Sonsbeek et al. 2014) are needed to directly compare different characteristics of feedback (e.g., intensity, frequency, tools) in order to examine what dose of outcome feedback is most beneficial to treatment effectiveness. This would also provide greater consistency across studies, which would make performing meta-analysis possible.

Furthermore, included studies had methodological limitations. For example, nearly half of the studies were underpowered and a significant number of studies suffered from incomplete data due to high rates of attrition, with intention-to-treat analyses rarely conducted. Thus, researchers should make an effort to conduct studies with large samples able to detect small differences between conditions and to more commonly apply analyses accounting for high attrition rates. Trials were often not described in enough detail, with authors not providing information regarding the process of randomisation, allocation, or blinding of participants. Continued efforts to ensure robust and transparent reporting procedures are recommended (Rennie 2001). Finally, most studies were conducted with adult samples, particularly with students in outpatient settings; more research is needed to evaluate the effect of outcome feedback in different populations and settings, for instance with children and young people.

Notwithstanding the above limitations, the present study provides a systematic review of evidence of outcome feedback in mental health settings. The search was conducted using multiple search engines according to the criteria of the best practice (Centre for Reviews and Dissemination 2009). The search was enhanced by a careful review of grey literature (e.g., Opengrey), trials registers, and reference lists of relevant literature and was conducted according to the guidelines of the Cochrane Review’s Handbook (Higgins et al. 2011) and PRISMA statement (Moher et al. 2009).

Our review provides robust evidence informing clinical practice about potential benefits of using feedback. The evidence is encouraging for clinicians to implement feedback interventions as it suggests that outcome feedback may result in higher levels of treatment effectiveness and treatment efficiency, especially for patients who are not-on-track and when it was provided to both clinicians and patients. Nonetheless, due to the heterogeneity of the methodology, feedback interventions, and included studies, clear conclusions cannot be drawn on the effects of outcome feedback on collaborative practice.

## Electronic supplementary material

Below is the link to the electronic supplementary material.

Supplementary material 1 (DOCX 15 kb)

Supplementary material 2 (DOCX 19 kb)
