# Supplementary material for: Feedback from Outcome Measures and Treatment Effectiveness, Treatment Efficiency, and Collaborative Practice: A Systematic Review
Source: Adm Policy Ment Health. 2016 Jan 7;43:325–43. doi: 10.1007/s10488-015-0710-5 (PMC4831994; doi:10.1007/s10488-015-0710-5)
Supplement: Supplementary file 1 — Supplementary material 1 (DOCX 15 kb) [file 10488_2015_710_MOESM1_ESM.docx]

| Supplementary Table 1.  *Reasons for exclusion of full-texts.* |  |
| --- | --- |
| Reasons for exclusion | *n* |
| Feedback provided only at baseline | 1 |
| Description of ROM system and/or feasibility, practicalities, or reliability of implementing ROM | 184 |
| Questionnaire validation study | 7 |
| Qualitative study | 12 |
| Non-mental health settings or outcomes | 27 |
| Duplicate | 9 |
| ROM used to explore the impact of an intervention | 12 |
| Review | 18 |
| Quality improvement study | 2 |
| Intervention did not meet criteria of ROM/feedback | 4 |
| Design was not RCT/CT | 15 |
| Total | 291 |

*Note.* ROM = routine outcome monitoring. RCT = randomised control trial. CT = control trial.
