# Supplementary material for: Feedback from Outcome Measures and Treatment Effectiveness, Treatment Efficiency, and Collaborative Practice: A Systematic Review
Source: Adm Policy Ment Health. 2016 Jan 7;43:325–43. doi: 10.1007/s10488-015-0710-5 (PMC4831994; doi:10.1007/s10488-015-0710-5)
Supplement: Supplementary file 2 — Supplementary material 2 (DOCX 19 kb) [file 10488_2015_710_MOESM2_ESM.docx]

| Supplementary Table 2.  *Assessment of risk of bias (adapted from Higgins & Green, 2011).* | | | |  |  |  |  |
| --- | --- | --- | --- | --- | --- | --- | --- |
| Reference | Sequence generation | Allocation concealment | Blinding of patients | Blinding of clinicians | Incomplete outcome data | Selective outcome reporting | Other sources of bias |
| Anker et al. (2009) | + | + | + | - | ? | + | + |
| Ashaye et al. (2003) | + | + | ? | ? | ? | + | + |
| Bickman et al. (2011) | ? | + | + | ? | + | + | - |
| Brodey et al. (2005) | ? | ? | ? | ? | - | + | - |
| Copeland (2007) | - | ? | ? | - | - | + | - |
| De Jong et al. (2012) | ? | ? | ? | ? | - | + | - |
| Galvinhill (2001) | ? | ? | ? | ? | - | + | - |
| Harmon et al. (2007) | ? | ? | + | - | + | + | + |
| Hawkins et al. (2004) | ? | ? | ? | - | + | + | - |
| Lambert et al. (2001) | ? | ? | ? | - | - | + | + |
| Lambert et al. (2002) | - | - | ? | - | - | + | - |
| Lester (2012) | ? | ? | ? | ? | - | + | + |
| Marshall et al. (2004) | + | + | ? | ? | ? | + | - |
| Murphy et al. (2012) | + | + | ? | - | ? | + | - |
| Newnham et al. (2010)/(Byrne et al., 2012) | - | - | ? | ? | ? | + | + |
| Cisneros (2010) | ? | ? | ? | ? | + | + | + |
| Priebe et al. (2007) | + | + | + | - | + | + | - |
| Probst et al. (2013) | ? | ? | ? | ? | - | + | + |
| Probst et al. (2014) | ? | ? | ? | ? | - | + | + |
| Puschner et al. (2009) | ? | ? | - | - | + | + | - |
| Reese, Norsworthy et al. (2009) | ? | ? | ? | - | - | + | - |
| Reese, Usher et al. (2009) | - | - | ? | - | ? | + | + |
| Reese et al. (2010) | ? | ? | ? | - | + | + | - |
| Reeves (2009) | - | - | ? | ? | ? | + | + |
| Rise et al. (2012) | + | - | - | - | + | + | - |
| Simon et al. (2012) | ? | ? | ? | - | ? | + | + |
| Simon et al. (2013) | ? | ? | ? | - | + | + | + |
| Slade et al. (2008)/Slade (2008) | ? | ? | ? | ? | ? | + | + |
| Soeken et al. (1981) | ? | ? | ? | - | ? | + | - |
| Trudeau (2001) | + | + | ? | ? | - | + | - |
| Truitt (2011) | ? | ? | ? | - | + | + | - |
| Whipple et al. (2003) | ? | ? | ? | - | ? | + | - |
| *Note. “*+” = low risk of bias; “-“ = high risk of bias; “?” = unknown risk of bias. | | | | | | | |
